# A network meta-analysis for toxicity of eight chemotherapy regimens in the treatment of metastatic/advanced breast cancer

**DOI:** 10.18632/oncotarget.13023

**Published:** 2016-11-02

**Authors:** Xiao-Hua Zhang, Shuai Hao, Bo Gao, Wu-Guo Tian, Yan Jiang, Shu Zhang, Ling-Ji Guo, Dong-Lin Luo

**Affiliations:** ^1^ Department of Breast, Thyroid Surgery, Research Institute of Surgery, Daping Hospital, Third Military Medical University, Chongqing 400042, P.R. China

**Keywords:** metastatic/advanced breast cancer, chemotherapy, toxicity, randomized controlled trials, bayesian network model

## Abstract

**Objective:**

To compare the incidence of toxicity of 8 different chemotherapy regimens, including doxorubicin + paclitaxel, doxorubicin, capecitabine, CMF (cyclophosphamide + methotrexate + 5-fluorouracil), FAC (fluorouracil + doxorubicin + cyclophosphamide), doxorubicin + docetaxel, doxorubicin + cyclophosphamide and paclitaxel in the treatment of metastatic/advanced breast cancer.

**Results:**

This network meta-analysis included 8 randomized controlled trials (RCTs). The findings revealed that, with regard to capecitabine alone regimen exhibited higher incidence of nausea/vomiting than doxorubicin + paclitaxel regimen, doxorubicin alone regimen and paclitaxel alone regimen in the treatment of patients with metastatic/advanced breast cancer (OR = 32.48, 95% CI = 1.65~2340.57; OR = 22.75, 95% CI = 1.03~1923.52; OR = 59.63, 95% CI = 2.22~5664.88, respectively). Furthermore, doxorubicin + cyclophosphamide regimen had lower incidence of febrile neutropenia than doxorubicin + docetaxel (OR = 0.17, 95% CI = 0.03~0.96). No significant difference in the incidence of stomatitis was observed among eight chemotherapy regimens.

**Materials and Methods:**

We initially searched PubMed, Cochrane Library and Embase databases from the founding of these databases to January 2016. Eligible studies investigating the 8 different chemotherapy regimens for treatment of metastatic/advanced breast cancer were included for direct and indirect comparison. The odds ratio (OR) and surface under the cumulative ranking curves (SUCRA) value of the incidence of toxicity among eight chemotherapy regimens were analyzed.

**Conclusions:**

Capecitabine alone regimen and doxorubicin + docetaxel regimen may have a more frequent toxicity in the treatment of metastatic/advanced breast cancer.

## INTRODUCTION

Breast cancer, one of the most common malignancies worldwide, comprises 3% of all cancers in women worldwide [1]. Approximately 1.7 million women were diagnosed with breast cancer and 522,000 women were died of this disease in 2012 [2]. In China, the incidence rate of breast cancer rose by five times from 1980 to 2011, from 6.4/100,000 to 31.93/100,000 [3]. The 5-year survival rates of patients with breast cancer were 83.3%, respectively 97.1% in stage I but only 24.5% in stage IV [4]. Lack of effective treatments against invasion and metastasis of breast cancer made it more difficult to increase the survival rate and quality of life in breast cancer patients [5]. Therefore, it is of great importance to identifying novel drugs/compounds with an anti-invasive potential, helping to control the metastasis of cancer cells, and hopefully, searching for novel anticancer drugs or compounds.

At present, chemotherapy plays a crucial part in the comprehensive treatment of breast cancer and various types of drugs are implied in the therapy, including docetaxel which can modify tumor phenotype, making tumor cells more amenable to T cell-mediated killing [6]. Paclitaxel has been reported to have a wide variety of anti-tumor activity following *in vivo* screens in laboratory mice implanted tumors and also found to possess cytotoxic activity in clinical application [7]. Also, doxorubicin is shown to trigger dose-dependent cardiotoxicity by redox cycling as well as the generation of reactive oxygen species [8]. A previous study has demonstrated that docetaxel combined with doxorubicin can double the clinical complete response rate for breast cancer patients diagnosed with negative axillary nodes [9]. Gemcitabine, an antimetabolite drug and a strong and specific deoxycytidine analog, have antitumor activity and tolerability in pancreatic cancer, lung cancer, ovarian cancer, as well as metastatic breast cancer [10, 11]. In addition, different combinations of drugs were often used to strengthen the anti-cancer effects and reduce the side effects. For instance, doxorubicin and cyclophosphamide combination therapy, one of various optional choices, being widespread used in patients with an indication for chemotherapy, is used as an effective therapy for early-stage breast cancer [12]. Moreover, cyclophosphamide, a common anticancer drug for breast tumor, was often used as a compound with other drugs, including cyclophosphamide + methotrexate + 5-fluorouracil (CMF), fluorouracil + doxorubicin + cyclophosphamide (FAC), Doxorubicin + Cyclophosphamide [13]. However, the optimal regime for advanced breast cancer remains undetermined.

In the last decade, network meta-analysis of randomized controlled trials (RCTs) whose advantage lie in facilitating indirect comparisons of multiple interventions, has been introduced as an extension of pairwise meta-analysis [14]. Unlike traditional meta-analysis, network meta-analysis is capable of indirect comparison not only by utilizing a common comparator to avoid the embarrassment of head-to-head experiment failure but also uniting both direct and indirect comparisons simultaneously [15, 16]. In this network meta-analysis, we aimed to compare the incidence of toxicity of different chemotherapy regimens in the treatment of metastatic/advanced breast cancer.

## RESULTS

### Characteristics of included studies

**Figure 1:**
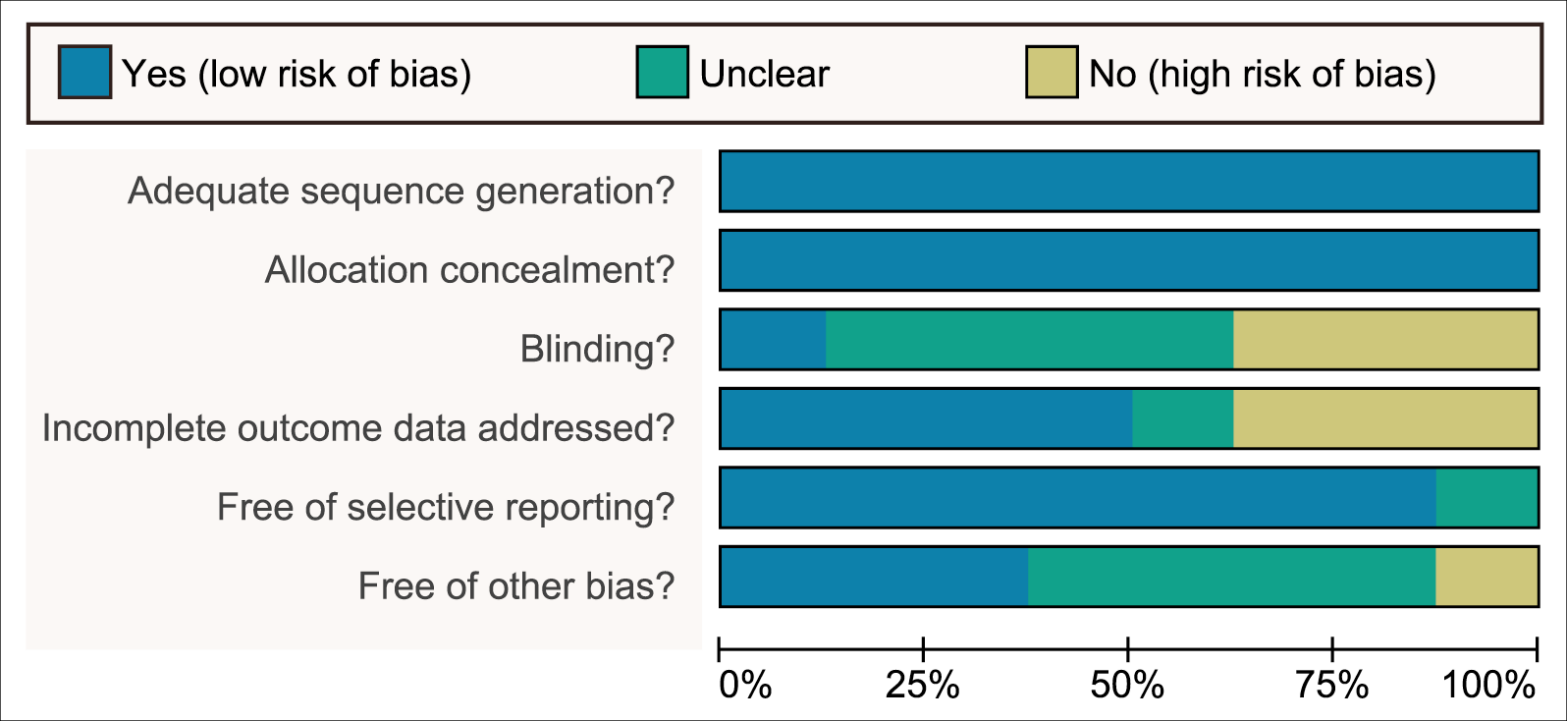
More than 2 reviewers evaluated the quality of randomized controlled trial with a modified Cochrane risk of bias assessment tool

A total of 2155 publications were initially retrieved in this study, and 133 for repeated assays, 157 for letters or summaries, 320 for non-human studies, and 319 for non-English papers were eliminated. Moreover, 455 for non-RCT studies, 248 articles unrelated to breast cancer, 512 articles unrelated to chemotherapy, 3 articles without data or incomplete data were also rejected from the rest of 1226 assays. Finally, eight RCTs met the inclusion criteria and were selected into our meta-analysis from 2001 to 2014 [17–24] (Supplementary Figure S1). A total of 2218 patients with metastatic/advanced breast cancer were recruited into meta-analysis, among which the majority of patients were treated with doxorubicin + paclitaxel chemotherapy regimen. Seven RCTs were conducted in the Caucasians, and the other RCT was conducted in the Asians. Furthermore, seven included studies were conducted by two-arm trial, and the rest 1 by three-arm trial. The characteristics of included studies were summarized in Table 1 and Cochrane risk of bias assessment in Figure 1.

**Table 1:** The baseline characteristics for included studies

First author	Year	Country	Interventions			Sample size				Age (years)			Disease stage	Median follow-up (months)
			T1	T2	T3	Total	T1	T2	T3	T1	T2	T3		
Smorenburg CH	2014	Netherlands	B	C		78	40	38		—	—		Stage IV	39
Leone JP	2014	America	D	E		126	62	64		54 (31–74)	51 (24–75)		Stage III	54
Stockler MR	2011	Australia	C	D		216	107	109		—	—		Stage IV	39.6
Cassier PA	2008	France	A	F		210	103	107		58 (32–79)	56 (32–79)		Stage IV	50.2
Evans TR	2005	UK	F	G		363	183	180		49 (27–74)	48 (25–73)		Stage IV	32
Sledge GW	2003	India	A	B	H	683	230	224	229	56 (27–76)	56 (27–78)	56(25–79)	Stage IV	26
Biganzoli L	2002	France	A	G		275	138	137		52 (29–70)	54 (28–70)		Stage IV	29.2
Jassem J	2001	Poland	A	E		267	134	133		50 (33–70)	50 (24–74)		Stage IV	29

Notes: T = Treatment; M = male; F = female; A = Doxorubicin + Paclitaxel; B = Doxorubicin; C = Capecitabine; D = CMF (cyclophosphamide + methotrexate + 5-fluorouracil); E = FAC (fluorouracil + doxorubicin + cyclophosphamide); F = Doxorubicin + Docetaxel; G = Doxorubicin + Cyclophosphamide; H = Paclitaxel.

### Pairwise meta-analysis

We conducted a direct comparison of the incidence of toxicity of 8 chemotherapy regimens during the treatment of metastatic/advanced breast cancer, and the results were as follows: in terms of nausea/vomiting, doxorubicin + docetaxel chemotherapy showed higher incidence of toxicity compared with doxorubicin + cyclophosphamide chemotherapy (OR = 3.38, 95%CI = 1.80~6.34); the incidence of toxicity was correspondingly higher in the patients undergoing doxorubicin chemotherapy than paclitaxel chemotherapy (OR = 2.67, 95%CI = 1.02~7.00); when compared with doxorubicin + cyclophosphamide and FAC chemotherapy, doxorubicin + paclitaxel chemotherapy delivered a lower incidence of toxicity (OR = 0.33, 95%CI = 0.15~0.73; OR = 0.39, 95%CI = 0.18~0.82, respectively). Concerning stomatitis, the incidence of toxicity of CMF chemotherapy was stronger than FAC chemotherapy (OR = 10.70, 95%CI = 1.31~87.19). Regarding febrile neutropenia, the incidence of toxicity of doxorubicin + docetaxel chemotherapy was higher than doxorubicin + cyclophosphamide chemotherapy (OR = 3.58, 95%CI = 2.10~6.11); the incidence of toxicity of doxorubicin + paclitaxel chemotherapy was stronger in comparison with doxorubicin + cyclophosphamide chemotherapy (OR = 4.74, 95%CI = 2.37~9.49); the incidence of toxicity of doxorubicin + paclitaxel chemotherapy was lower than doxorubicin + docetaxel chemotherapy (OR = 0.29, 95%CI = 0.16~0.53) (Supplementary Table S1).

### Evidential network

**Figure 2:**
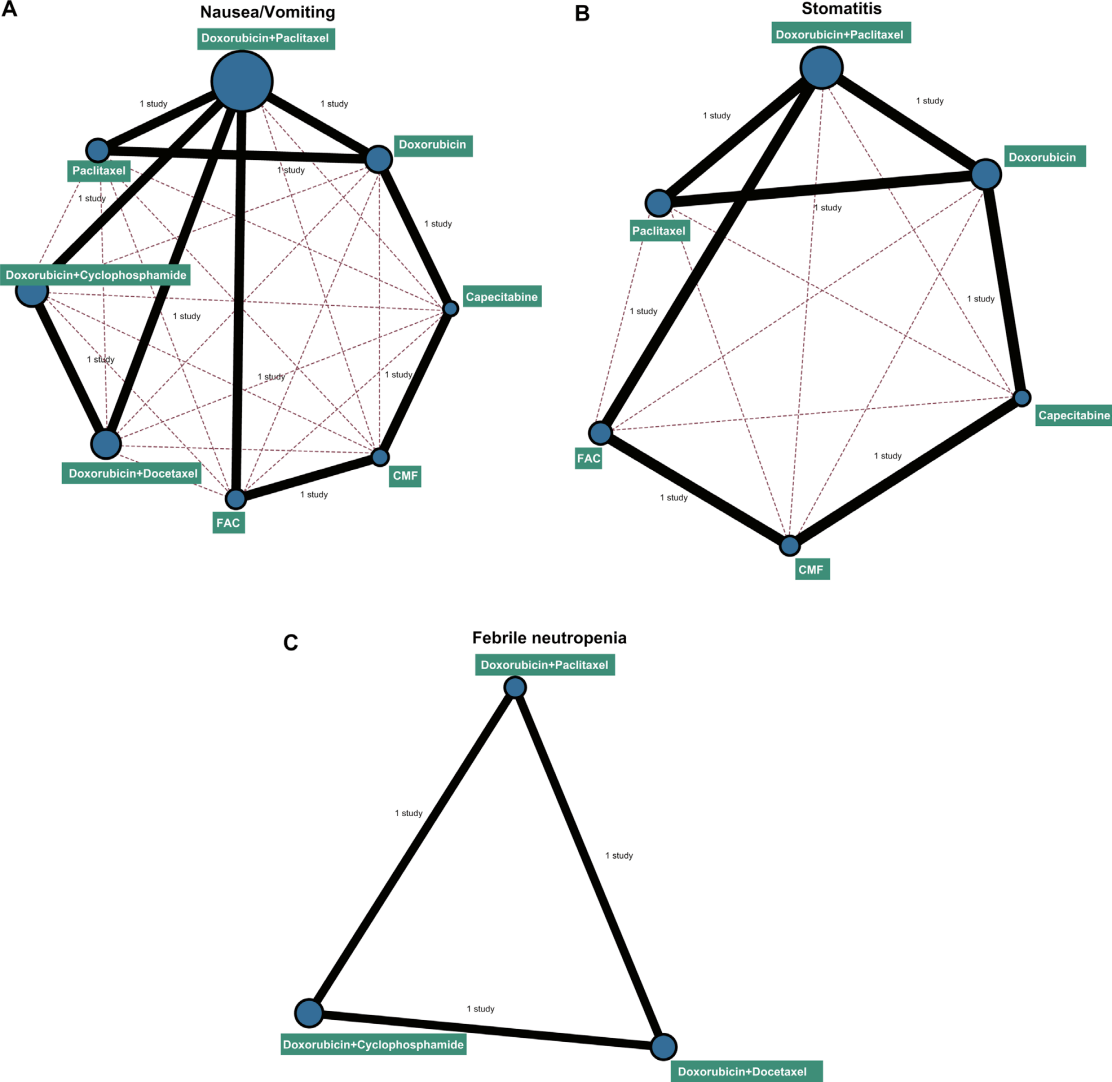
Network diagrams of the incidence of nausea/vomiting, stomatitis and febrile neutropenia Note: (**A**) nausea/vomiting; (**B**) stomatitis; (**C**) febrile neutropenia; CMF: cyclophosphamide + methotrexate + 5-fluorouraci; FAC: fluorouracil + doxorubicin + cyclophosphamide. The width of the lines is proportional to the number of trials comparing every pair of treatments, and the size of every circle is proportional to the number of randomly assigned participants (sample size).

Eight chemotherapy regimens were included in this study: doxorubicin + paclitaxel, doxorubicin, capecitabine, CMF (cyclophosphamide+methotrexate+5-fluorouraci), FAC (fluorouracil+doxorubicin+cyclophosphamide), doxorubicin + docetaxel, doxorubicin + cyclophosphamide, and paclitaxel. In terms of nausea/vomiting, stomatitis and febrile neutropenia, a largest number of patients received doxorubicin + paclitaxel chemotherapy, while the least number of patients received capecitabine chemotherapy regimen (Figure 2).

### Tests of discordancy

The nausea/vomiting, stomatitis and febrile neutropenia outcomes were analyzed by discordancy test with the node-splitting method, and the analysis showed that all outcomes of the direct and indirect evidence are consistent, thus the consistency model should be adopted (all *P* > 0.05) (Table 2).

**Table 2:** OR values and *P* values of direct and indirect pairwise comparisons of eight treatment modalities under three endpoint outcomes

Pairwise comparisons	Direct OR values			Indirect OR values			*P* values		
	Nau	Sto	Feb	Nau	Sto	Feb	Nau	Sto	Feb
B vs A	1.70	1.80	NR	0.003	11.0	NR	0.335	0.651	NR
E vs A	2.60	0.97	NR	4.30	0.20	NR	0.413	0.660	NR
F vs A	2.80	NR	3.50	1.40	NR	0.72	0.576	NR	0.246
G vs A	3.20	NR	0.21	6.40	NR	0.99	0.605	NR	0.270
C vs B	1.40	0.18	NR	12.0	0.77	NR	0.398	0.648	NR
D vs C	0.18	9.40	NR	0.004	57.00	NR	0.353	0.654	NR
E vs D	0.67	0.068	NR	0.004	0.360	NR	0.379	0.618	NR
G vs F	2.40	NR	0.27	1.20	NR	4.20	0.656	NR	0.264

Notes: NR = Not report; OR = odds ratio; Nau = Nausea/vomiting; Sto = Stomatitis; Feb = Febrile neutropenia; A = Doxorubicin + Paclitaxel; B = Doxorubicin; C = Capecitabine; D = CMF (cyclophosphamide + methotrexate + 5-fluorouracil); E = FAC (fluorouracil + doxorubicin + cyclophosphamide); F = Doxorubicin + Docetaxel; G = Doxorubicin + Cyclophosphamide; H = Paclitaxel.

### Network meta-analysis

**Figure 3:**
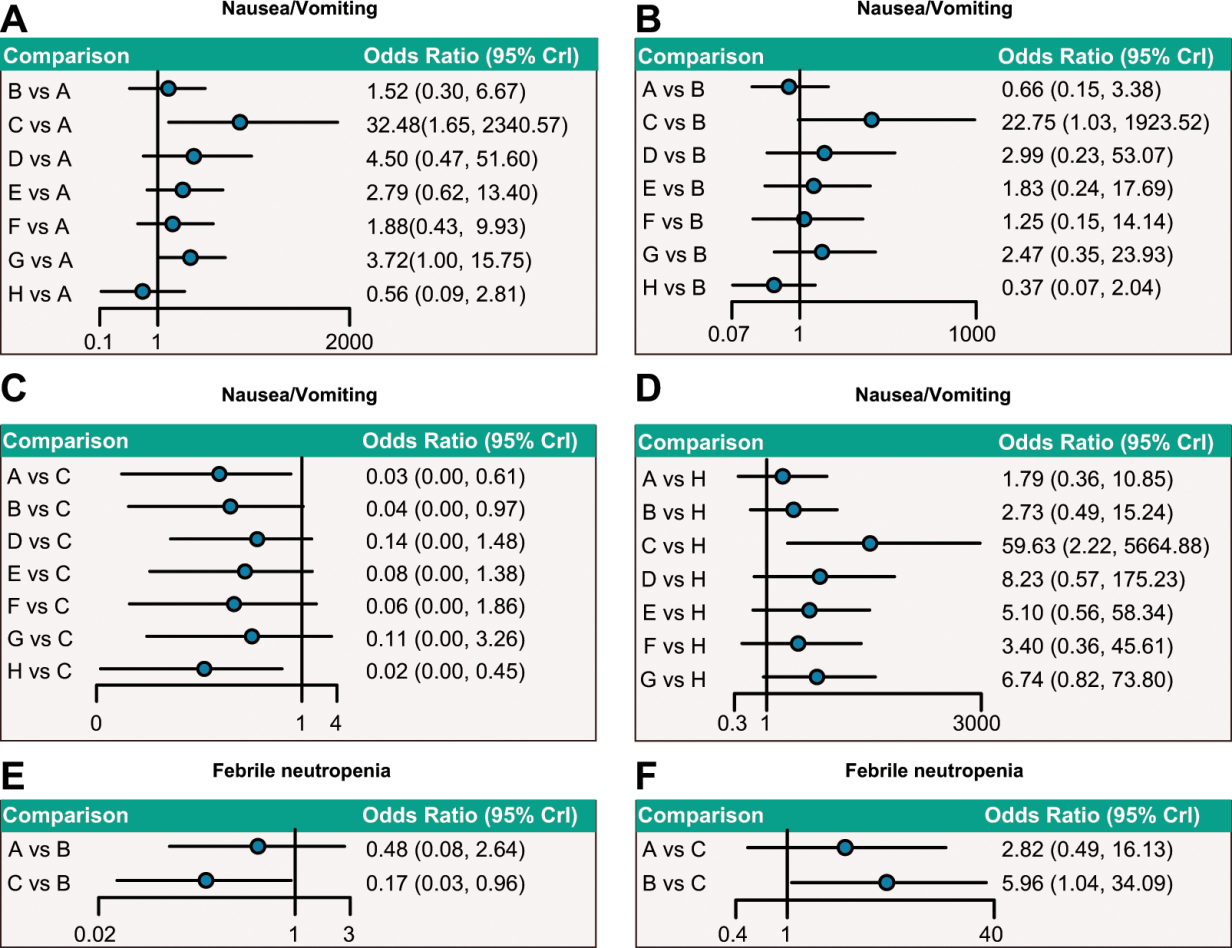
Relative network diagrams of the incidence of nausea/vomiting and febrile neutropenia Note: nausea/vomiting: A = doxorubicin + paclitaxel; B = doxorubicin; C = capecitabine; D = CMF (cyclophosphamide + methotrexate + 5-fluorouraci); E = FAC (fluorouracil + doxorubicin + cyclophosphamide); F = doxorubicin + docetaxel; G = doxorubicin + cyclophosphamide; H = paclitaxel; Ffebrile neutropenia: A = doxorubicin + paclitaxel; B = doxorubicin + docetaxel; C = doxorubicin + cyclophosphamide; CrI: Credible interval; The confidence interval of OR passes through 1, indicating no significance.

As Supplementary Table S2 and Figure 3 indicated, concerning nausea/vomiting, capecitabine chemotherapy exhibited higher toxicity when compared with doxorubicin + paclitaxel, doxorubicin, and paclitaxel chemotherapy regimens (OR = 32.48, 95%CI = 1.65~2340.57; OR = 22.75, 95%CI = 1.03~1923.52; OR = 59.63, 95%CI = 2.22, 5664.88, respectively). When compared with doxorubicin + docetaxel chemotherapy, the incidence of toxicity of doxorubicin + cyclophosphamide chemotherapy was lower with regard to febrile neutropenia (OR = 0.17, 95%CI = 0.03~0.96). However, no significance was shown in the incidence of toxicity of each chemotherapy regimen as for stomatitis.

### Cumulative probability

As shown in Table 3, the SUCRA values demonstrated that paclitaxel chemotherapy ranked the highest (92.3%), while capecitabine chemotherapy ranked the lowest (16.1%) with regard to nausea/vomiting. However capecitabine chemotherapy regimen ranked the highest (76.8%) and CMF chemotherapy ranked the lowest (26.2%) concerning stomatitis. As for febrile neutropenia, doxorubicin + cyclophosphamide chemotherapy ranked the highest (96.0%) and doxorubicin + docetaxel ranked the lowest (39.7%). In a meta-regression analysis to assess potential bias, no significant difference was found in the SUCRA values of eight chemotherapy regimens under three outcome measures after considering covariate (median follow-up: *P* = 0.314, disease stage: *P* = 0.980), thus the median follow-up and disease stage were not the main source of heterogeneity (Table 4)

**Table 3:** SUCRA values of eight treatment modalities under three endpoint outcomes

Treatments	SUCRA values		
	Nausea/vomiting	Stomatitis	Febrile neutropenia
**A**	0.820	0.612	0.647
**B**	0.673	0.413	NR
**C**	**0.161**	0.768	NR
**D**	0.399	**0.262**	NR
**E**	0.506	0.720	NR
**F**	0.623	NR	**0.397**
**G**	0.394	NR	0.960
**H**	0.923	0.720	NR

Notes: SUCRA = Surface under the cumulative ranking curves; NR = Not report; A = Doxorubicin + Paclitaxel; B = Doxorubicin; C = Capecitabine; D = CMF (cyclophosphamide + methotrexate + 5-fluorouracil); E = FAC (fluorouracil + doxorubicin + cyclophosphamide); F = Doxorubicin + Docetaxel; G = Doxorubicin + Cyclophosphamide; H = Paclitaxel.

**Table 4:** SUCRA values of eight chemotherapy regimens under three outcome measures after considering covariate

Treatments	SUCRA values (considering covariate)	
	Median follow-up	Disease stage
**A**	**0.793**	**0.798**
**B**	0.542	0.530
**C**	0.228	0.226
**D**	0.485	0.488
**E**	0.537	0.544
**F**	0.645	0.646
**G**	0.397	0.401
**H**	0.873	0.867

Notes: SUCRA = Surface under the cumulative ranking curves; A = Doxorubicin + Paclitaxel; B = Doxorubicin; C = Capecitabine; D = CMF (cyclophosphamide + methotrexate + 5-fluorouracil); E = FAC (fluorouracil + doxorubicin + cyclophosphamide); F = Doxorubicin + Docetaxel; G = Doxorubicin + Cyclophosphamide; H = Paclitaxel.

## DISCUSSION

After collecting historical cases and analyzing 8 different chemotherapy regimens for metastatic/advanced breast cancer by pairwise and network meta-analysis, we found evidence that capecitabine regimen showed stronger toxicity in the treatment of metastatic/advanced breast cancer, while the incidence of toxicity of doxorubicin + paclitaxel, doxorubicin, paclitaxel, doxorubicin + docetaxel regimens were relatively lower.

The analysis of pairwise and network meta-analysis consistently indicated that the incidence of toxicity of capecitabine regimen was higher than other regimens during the treatment of metastatic/advanced breast cancer. As an orally administered chemotherapy of fluorouracil, capecitabine shows antineoplasmic activity in various models, which is activated metabolically at the tumor site [25]. Besides, capecitabine is primarily transformed into 5′-deoxy-5-fluorocytidine in the liver, and further into 5′-deoxy-5-fluorouridine (5′-DFUR). Subsequently, 5′-DFUR is metabolized to active 5-FU by thymidine phosphorylase (TP), and higher concentration of TP is present in some tumor tissues in comparison with normal tissues [26, 27]. As several studies indicated, capecitabine treatment has confirmed its efficacy and activity in metastatic/advanced breast cancer [28–31]. However, hand-foot syndrome (HFS), a common side-effect and dose-limiting toxicity, often results from capecitabine treatment for patients with metastatic/advanced breast cancer, which seriously affects patients' daily activity and life quality [32, 33]. While the pathogenesis of HFS is not completely clear, which may be associated with enzymes involved in capecitabine metabolism, and the high activity of dihydropyrimidine dehydrogenas (DPD) and TP contribute to metabolite accumulation of capecitabine [34]. On the other hand, Saher et al. points out that capecitabine eliminates from the eccrine gland system, thus the increased number of eccrine glands in hand and foot may play a key role in HFS pathogenesis [35]. Meanwhile, Stockler et al. has proven that, although febrile neutropenia, infection, stomatitis, and serious adverse events were more common with CMF, HFS presented higher frequency on metastatic/advanced breast cancer receiving capecitabine treatment than CMF [19].

On the other hand, the pairwise and network meta-analysis also revealed that four chemotherapy regimens displayed lower toxicity in metastatic/advanced breast cancer patients (doxorubicin + paclitaxel, doxorubicin, paclitaxel, doxorubicin + docetaxel). As an anthracycline antibiotic, doxorubicin is one of the most effective chemotherapeutics widely used for the treatment of breast cancer [36]. Additionally, the polymer-doxorubicin can cause decreased plasma concentration by sustaining the release of anti-neoplastic drugs, which may reveal the association with the dose-limiting cardiotoxicity [37]. As studies reported, liposomal doxorubicin has exhibited diminished the cardiac toxicity in various clinical tests, and the combination of pegylated liposomal doxorubicin and other anti-tumor drugs was positive against metastatic/advanced breast cancer [38–40]. Belonging to taxane drugs category, paclitaxel specially binds to β-tubulin subunit of the N-terminal in the microtubule fasolculus and inhibits its growth and cell replication [7]. Furthermore, paclitaxel also shows the unique ability to restrain the proliferation and migration of tumor cells [41]. Hence, the lower toxicity of paclitaxel is reasonable due to its specific therapeutic targets. For patients with metastatic triple-negative breast cancer, cisplatin + gemcitabine regimen might be an alternative or even the favorable first-line chemotherapy strategy when compared with the more established paclitaxel + gemcitabine regimen [42]. For women with metastatic breast cancer, doxorubicin and paclitaxel as first-line therapy has an obvious advantage over FAC in the aspects of response rate, median time to progression, as well as in overall survival [43]. In elderly metastatic breast cancer patients, the first-line single-agent chemotherapy of both pegylated liposomal doxorubicin and capecitabine showed comparable efficacy and acceptable tolerance [17]. In terms of docetaxel, this chemotherapy possesses a variety of advantages on the treatment of metastatic/advanced breast cancer, but it is followed by severe side effects including anemia, neuropathy and asthenia, myelosuppression, and hypersensitivity reaction [44]. With the development of nanotechnology, the use of nanoparticle drug can prevent side effects following docetaxel and reduce docetaxel toxicity [45]. Interestingly, Sledge et al. confirms that single treatments of doxorubicin and paclitaxel regimens exhibit lower toxicity than doxorubicin + paclitaxel therapy [22]. As mentioned above, the lower toxicities of doxorubicin, paclitaxel and docetaxel have been reported, thus the interaction between the combination of doxorubicin + paclitaxel, and doxorubicin + docetaxel contribute to the less toxicity in breast cancer. Cassier PA et al. supported that in the treatment of metastatic breast cancer, the combinations of paclitaxel + doxorubicin and docetaxel + doxorubicin have shown superiority over other treatments; doxorubicin combined with docetaxel or paclitaxel have yield similar results in the treatment of metastatic breast cancer, but these two ways of combinations have induced different toxicities because of the specific toxicity profile [20].

In our study, we adapted the combined analysis of pairwise and network, and the application of the node-splitting method aimed to verify direct and indirect outcomes, which can compare a variety of interventions, thus more accurate and comprehensive conclusions were obtained [46]. However, it must note that the experiment was relatively single due to small included number of articles and lack of contrast between the cross-research projects, resulting in restriction of universal conclusion. Moreover, with regard to febrile neutropenia, the involved assays did not include doxorubicin, capecitabine, CMF, FAC, and paclitaxel chemotherapy, thus it fail to be analyzed in the calculation of SCRUA value. And nausea/vomiting, the amount of interventions related to nausea/vomiting, stomatitis and febrile neutropenia outcomes were not equal, so the cluster analysis fails to be in progress, which may result in the appearance of subtle bias. Furthermore, other side effects were not included in the study because of insufficient enrolled literatures and chemotherapy incapable of forming rings. Nevertheless, major cases are enrolled in the study, and consistent with the research progress, hence the conclusion has certain value and significance.

In conclusion, these results indicate that capecitabine alone regimen and doxorubicin + docetaxel regimen may have a more frequent toxicity in the treatment of metastatic/advanced breast cancer, which has a certain guiding significance for the clinical use and treatment of metastatic/advanced breast cancer.

## MATERIALS AND METHODS

### Search strategy

Computer-based retrieval in PubMed, the Cochrane Library, and Embase English databases (from inception to January 2016), combined with manual retrieval of related references were performed. Combining the keywords and free words, the searches terms were as followed: chemotherapy, cyclophosphamide, doxorubicin, docetaxel, capecitabine, gemcitabine, advanced breast cancer, as well as RCT.

### Pubmed search strategy

#1: “breast neoplasms”[mh] OR breast cancer[tiab] OR Neoplasm, Breast[tiab] OR Tumors, Breast OR Mammary Neoplasms, Human[tiab] OR Human Mammary Neoplasm[tiab] OR Mammary Carcinoma, Human[tiab] OR Carcinomas, Human Mammary[tiab] OR Mammary Carcinomas, Human[tiab] OR Mammary Cancer[tiab] OR Cancer of Breast[tiab]

#2: chemotherapy[tiab] OR “Cyclophosphamide” [mh] OR “Doxorubicin”[mh] OR Farmiblastina[tiab] OR “Paclitaxel”[mh] OR “Docetaxel”[mh] OR “Capecitabine” [mh] OR “gemcitabine” [mh] OR “Epothilone”[mh] OR “vinorelbine”[Supplementary Concept] OR “DDP” [Supplementary Concept] OR “Carboplatin” [Supplementary Concept] OR “oxaliplatin”[Supplementary Concept] OR “Pirarubicin”[Supplementary Concept] OR “Epirubicin”[mh]

#3: “randomized controlled trial”[pt] OR “controlled clinical trial”[pt] OR “randomized controlled trials as topic”[Mesh] OR “clinical trials as topic”[mh] OR “controlled clinical trials as topic”[mh] OR placebos[mh] OR “random allocation”[mh] OR “double-blind method”[mh] OR randomized[tiab] OR placebo[tiab] OR randomization[tiab] OR randomly allocated[tiab] OR ((double[tw] OR treble[tw] OR triple[tw]) AND (mask* [tw] OR blind* [tw]))

#4: #1 and #2 and #3

### Selection criteria

The inclusion criteria: (1) study type: RCT; (2) interventions: doxorubicin + paclitaxel, doxorubicin, capecitabine, CMF (cyclophosphamide + methotrexate + fluorouraci), FAC (fluorouracil + doxorubicin + cyclophosphamide), doxorubicin + docetaxel, doxorubicin + cyclophosphamide, and paclitaxel; (3) chemotherapy: first-line therapy; (4) disease stage: stage III or IV; (5) patient population: hospital-based patient; (6) subjects: at least one measurable lesion in patients with metastatic/advanced breast cancer according to the Response Evaluation Criteria in Solid Tumors (RECIST) [47]; (7) outcomes: patients with nausea/vomiting, stomatitis, and febrile neutropenia. The exclusion criteria: (1) breast cancer patients with inflammatory reaction; (2) patients undergoing systemic and radiation therapy; (3) patients with other serious diseases or mental illness history; (4) patients with chronic liver disease; (5) incomplete literature data (for example: non-matched pair study); (6) non RCT; (7) duplications; (8) conference report, systems assessment and abstract; (9) non-English literature; (10) non-human study.

### Data extraction and quality assessment

Two reviewers abstracted the data independently with the unified data collection form, and disagreements were resolved by discussion with a number of investigators. The trial size, trial design, details of intervention including treatment duration, patient characteristics such as sex, age, mean duration of follow-up time as well as outcome data were extracted. For crossover trials, the data was extracted from the first period only in order to avoid possible carryover effects. The means and measures of dispersion were approximated from figures in the reports as previously described whenever necessary. Whenever possible, we extracted results from the intention-to-treat analyses. More than 2 reviewers evaluated the quality of RCT with a modified Cochrane risk of bias assessment tool [48], which included the following 6 domains: random assignment, allocation concealment, blinding of participants, incomplete outcome data, selective outcome reporting, and other sources of bias. The RCT assessment was explicitly evaluated as “yes,” “no,” or “unclear” for each domain to assign a low, high, or unclear risk of bias, respectively. The study was regarded with low risk of bias when one or no domain was defined as “unclear” or “no”, the study was regarded with high risk of bias when four or more domains were defined as “unclear” or “no”, and the study was regarded with moderate risk of bias when two or three domains were defined as “unclear” or “no” [49]. Quality assessment and investigation of publication bias were conducted by Review Manager 5 (RevMan 5.2.3, Cochrane Collaboration, Oxford, UK).

### Statistical methods

Firstly, pairwise meta-analyses of direct evidence were conducted by the fixed-effects model, with R version 3.2.1 and the meta-package. We also calculated the pooled results of odd ratios (OR) with 95% credible intervals (CrIs) of three end point outcomes. Chi-square test and I-square test were carried out for testing heterogeneity among the studies [50]. Then, R version 3.2.1 and network package were used for drawing the network graph. Besides, each node represented a variety of interventions, the node size represented the sample size, and the lines between the nodes represented the included numbers of research. Also, we implemented a random-effects network meta-analysis with the gemtc package, which modeled the relative effects (e.g.log-odds ratio) responding to a generalized linear model (GLM) under the Bayesian framework by connecting to JAGS, OpenBUGS or WinBUGS as first described by Lu and Ades [51], and improved by others [52, 53]. Subsequently, we also adopted the node-splitting method to evaluate the consistency between direct and indirect evidence, and chosen the consistency or inconsistency model based on the results. If the node segmentation showed that the *P value* was higher than 0.05, the consistency model was used to analyze the results [54]. In order to facilitate the interpretation of ORs, we calculated the chance of each intervention that was the optimal treatment method on the basis of a Bayesian approach using probability values summarized as surface under the cumulative ranking curve (SUCRA). The higher the SUCRA value, the better the rank of the intervention [55, 56]. R 3.2.1 was conducted for all analyses.

## SUPPLEMENTARY MATERIALS FIGURES AND TABLES


