# Supplementary material for: A network meta-analysis for toxicity of eight chemotherapy regimens in the treatment of metastatic/advanced breast cancer
Source: Oncotarget. 2016 Nov 2;7(51):84533–43. doi: 10.18632/oncotarget.13023 (PMC5356679; doi:10.18632/oncotarget.13023)
Supplement: Supplementary file 1 [file oncotarget-07-84533-s001.pdf]

# A network meta-analysis for toxicity of eight chemotherapy regimens in the treatment of metastatic/advanced breast cancer

## Supplementary Materials

**Supplementary Table S1: Assessment of heterogeneity for direct comparisons and estimated OR and 95%CI from pairwise meta-analysis**

| Included studies | Comparisons | Toxicity events | | Pairwise meta-analysis | | |
|---|---|---|---|---|---|---|
| | | Treatment1 | Treatment2 | OR (95%CI) | $I^2$ | $P_h$ |
| **Nausea/vomiting** | | | | | | |
| 1 study | B VS. C | 0/40 | 1/38 | 0.31 (0.01; 7.81) | NA | NA |
| 1 study | D VS. E | 8/62 | 6/64 | 1.43 (0.47; 4.40) | NA | NA |
| 1 study | C VS. D | 8/107 | 4/109 | 2.12(0.62; 7.27) | NA | NA |
| 1 study | A VS. F | 2/103 | 5/107 | 0.40 (0.08; 2.13) | NA | NA |
| 1 study | F VS. G | 43/183 | 15/180 | **3.38 (1.80; 6.34)** | NA | NA |
| 1 study | A VS. B | 10/230 | 15/224 | 0.63 (0.28;1.44) | NA | NA |
| 1 study | B VS. H | 15/224 | 6/229 | **2.67 (1.02;7.00)** | NA | NA |
| 1 study | A VS. H | 10/230 | 6/229 | 1.69 (0.60;4.73) | NA | NA |
| 1 study | A VS. G | 9/136 | 24/135 | **0.33 (0.15; 0.73)** | NA | NA |
| 1 study | A VS. E | 11/134 | 25/133 | **0.39 (0.18; 0.82)** | NA | NA |
| **Stomatitis** | | | | | | |
| 1 study | B VS. C | 4/40 | 1/38 | 4.11 (0.44;38.57) | NA | NA |
| 1 study | D VS. E | 9/62 | 1/64 | **10.70 (1.31; 87.19)** | NA | NA |
| 1 study | C VS. D | 1/107 | 6/109 | 0.16 (0.02;1.37) | NA | NA |
| 1 study | A VS. B | 10/230 | 17/224 | 0.55 (0.25;1.24) | NA | NA |
| 1 study | A VS. E | 1/134 | 1/133 | 0.99 (0.06; 16.03) | NA | NA |
| **Febrile neutropenia** | | | | | | |
| 1 study | A VS. F | 22/103 | 52/107 | **0.29 (0.16; 0.53)** | NA | NA |
| 1 study | F VS. G | 63/183 | 23/180 | **3.58 (2.10; 6.11)** | NA | NA |
| 1 study | A VS. G | 43/136 | 12/135 | **4.74( 2.37; 9.49)** | NA | NA |

Notes: 95%CI = 95%confidence intervals; OR = odds ratio; NA = not available; A = Doxorubicin + Paclitaxel; B = Doxorubicin; C = Capecitabine; D = CMF (cyclophosphamide + methotrexate + 5-fluorouracil); E = FAC (fluorouracil + doxorubicin + cyclophosphamide); F = Doxorubicin + Docetaxel; G = Doxorubicin + Cyclophosphamide; H = Paclitaxel.

## Supplementary Table S2: Odds ratio and 95% confidence intervals of eight drugs in the treatment of metastatic/advanced breast cancer in terms of nausea/vomiting, stomatitis and febrile neutropenia

| OR (95%CI) | | | | | | | |
|---|---|---|---|---|---|---|---|
| **Nausea/vomiting** | | | | | | | |
| **A** | 1.52 (0.30, 6.67) | **32.48 (1.65, 2340.57)** | 4.50 (0.47, 51.60) | 2.79 (0.62, 13.40) | 1.88 (0.43, 9.93) | 3.72 (1.00, 15.75) | 0.56 (0.09, 2.81) |
| 0.66 (0.15, 3.38) | **B** | **22.75 (1.03, 1923.52)** | 2.99 (0.23, 53.07) | 1.83 (0.24, 17.69) | 1.25 (0.15, 14.14) | 2.47 (0.35, 23.93) | 0.37 (0.07, 2.04) |
| 0.03 (0.00, 0.61) | 0.04 (0.00, 0.97) | **C** | 0.14 (0.00, 1.48) | 0.08 (0.00, 1.38) | 0.06 (0.00, 1.86) | 0.11 (0.00, 3.26) | 0.02 (0.00, 0.45) |
| 0.22 (0.02, 2.12) | 0.33 (0.02, 4.38) | 6.99 (0.68, 287.19) | **D** | 0.62 (0.10, 3.38) | 0.40 (0.03, 6.87) | 0.81 (0.05, 12.31) | 0.12 (0.01, 1.77) |
| 0.36 (0.07, 1.61) | 0.55 (0.06, 4.09) | 11.82 (0.73, 689.98) | 1.62 (0.30, 9.81) | **E** | 0.66 (0.08, 6.77) | 1.31 (0.17, 11.49) | 0.20 (0.02, 1.80) |
| 0.53 (0.10, 2.32) | 0.80 (0.07, 6.75) | 18.08 (0.54, 1641.97) | 2.47 (0.15, 37.88) | 1.52 (0.15, 12.91) | F | 1.97 (0.48, 7.51) | 0.29 (0.02, 2.74) |
| 0.27 (0.06, 1.00) | 0.40 (0.04, 2.88) | 9.09 (0.31, 723.11) | 1.23 (0.08, 18.81) | 0.76 (0.09, 5.86) | 0.51 (0.13, 2.08) | **G** | 0.15 (0.01, 1.22) |
| 1.79 (0.36, 10.85) | 2.73 (0.49, 15.24) | **59.63 (2.22, 5664.88)** | 8.23 (0.57, 175.23) | 5.10 (0.56, 58.34) | 3.40 (0.36, 45.61) | 6.74 (0.82, 73.80) | **H** |
| **Stomatitis** | | | | | | | |
| **A** | 2.01 (0.21, 23.33) | 0.50 (0.02, 17.99) | 6.84 (0.17, 357.56) | 0.65 (0.02, 18.33) | 0.67 (0.06, 8.90) | | |
| 0.50 (0.04, 4.82) | **B** | 0.25 (0.01, 4.33) | 3.43 (0.08, 172.19) | 0.32 (0.01, 11.38) | 0.34 (0.03, 3.43) | | |
| 2.00 (0.06, 59.23) | 4.02 (0.23, 90.30) | **C** | 13.11 (0.75, 428.91) | 1.23 (0.03, 51.54) | 1.37 (0.03, 50.54) | | |
| 0.15 (0.00, 5.90) | 0.29 (0.01, 12.96) | 0.08 (0.00, 1.33) | **D** | 0.09 (0.00, 1.45) | 0.10 (0.00, 6.51) | | |
| 1.54 (0.05, 58.75) | 3.14 (0.09, 164.01) | 0.81 (0.02, 29.98) | 10.95 (0.69, 233.50) | **E** | 1.05 (0.02, 71.77) | | |
| 1.50 (0.11, 17.48) | 2.97 (0.29, 38.54) | 0.73 (0.02, 29.71) | 10.27 (0.15, 756.19) | 0.95 (0.01, 43.89) | **H** | | |
| **Febrile neutropenia** | | | | | | | |
| **A** | 2.09 (0.38, 12.26) | 0.35 (0.06, 2.02) | | | | | |
| 0.48 (0.08, 2.64) | **B** | 0.17 (0.03, 0.96) | | | | | |
| 2.82 (0.49, 16.13) | 5.96 (1.04, 34.09) | **C** | | | | | |

Note: OR = odds ratio; CI = confidence interval; A = doxorubicin + paclitaxel; B = doxorubicin; C = capecitabine; D = CMF (cyclophosphamide + methotrexate + 5-fluorouraci); E = FAC (fluorouracil + doxorubicin + cyclophosphamide); F = doxorubicin + docetaxel; G = doxorubicin + cyclophosphamide; H = paclitaxel.

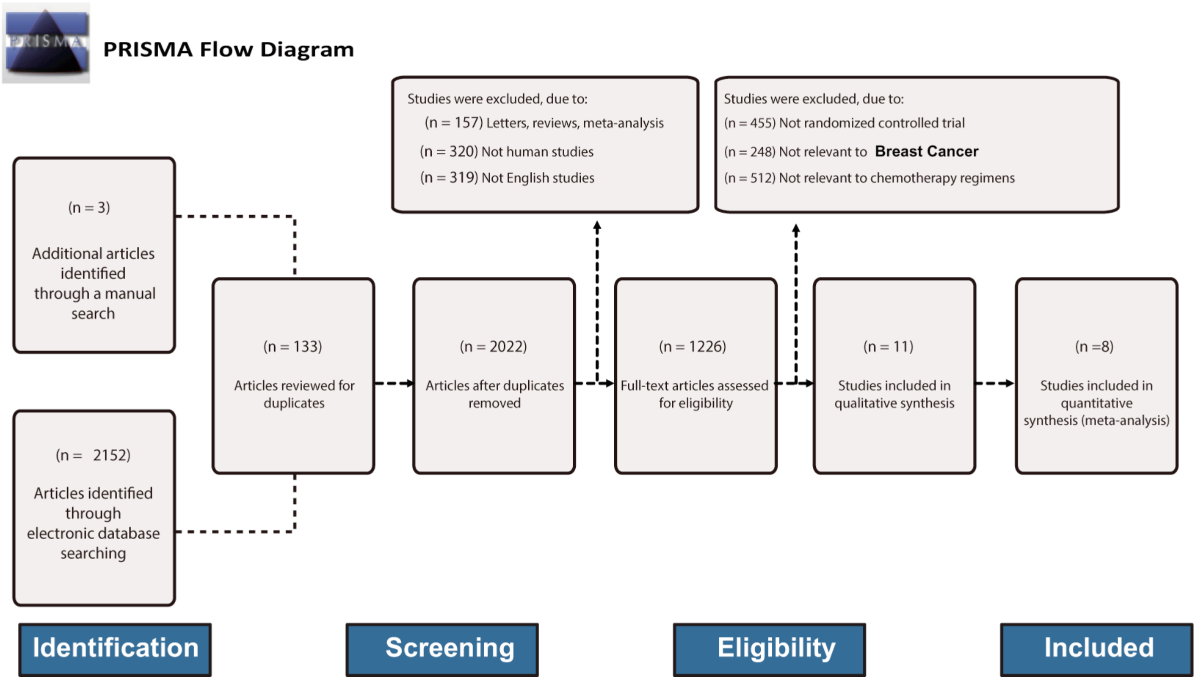


**Supplementary Figure S1: PRISMA statement flow chart shows the detailed procedures of study screening and exclusion reasons.** Eight randomized controlled trials were included in this network meta-analysis, a total of 2218 patients with metastatic/advanced breast cancer were recruited into meta-analysis.
